# Salzburg Intensive Care database (SICdb): a detailed exploration and comparative analysis with MIMIC-IV

**DOI:** 10.1038/s41598-024-61380-0

**Published:** 2024-05-20

**Authors:** Sina Sadeghi, Lars Hempel, Niklas Rodemund, Toralf Kirsten

**Affiliations:** 1 Department for Medical Data Science, Leipzig University Medical Center, Leipzig, Germany; 2 Institute for Medical Informatics, Statistics and Epidemiology, Leipzig University, Leipzig, Germany; 3 Faculty Applied Computer and Bio Sciences, Mittweida University of Applied Sciences, Mittweida, Germany; 4 Department of Anaesthesiology, Perioperative Medicine and Intensive Care Medicine, Paracelsus Medical University of Salzburg, Salzburg, Austria

**Keywords:** Intensive care unit, Salzburg Intensive Care database, Data science, Machine learning, Health care, Medical research, Mathematics and computing

## Abstract

The utilization of artificial intelligence (AI) in healthcare is on the rise, demanding increased accessibility to (public) medical data for benchmarking. The digitization of healthcare in recent years has facilitated medical data scientists’ access to extensive hospital data, fostering AI-based research. A notable addition to this trend is the Salzburg Intensive Care database (SICdb), made publicly available in early 2023. Covering over 27 thousand intensive care admissions at the University Hospital Salzburg from 2013 to 2021, this dataset presents a valuable resource for AI-driven investigations. This article explores the SICdb and conducts a comparative analysis with the widely recognized Medical Information Mart for Intensive Care - version IV (MIMIC-IV) database. The comparison focuses on key aspects, emphasizing the availability and granularity of data provided by the SICdb, particularly vital signs and laboratory measurements. The analysis demonstrates that the SICdb offers more detailed information with higher data availability and temporal resolution for signal data, especially for vital signs, compared to the MIMIC-IV. This is advantageous for longitudinal studies of patients’ health conditions in the intensive care unit. The SICdb provides a valuable resource for medical data scientists and researchers. The database offers comprehensive and diverse healthcare data in a European country, making it well suited for benchmarking and enhancing AI-based healthcare research. The importance of ongoing efforts to expand and make public datasets available for advancing AI applications in the healthcare domain is emphasized by the findings.

## Introduction

With the rapid increase in the use of artificial intelligence (AI) in healthcare, the open publication of medical datasets has become crucial for promoting data-driven clinical research ^1,2^. The continued advancement of AI has generally benefited greatly from the availability of adequate computing power, including the adoption of graphics processing units (GPUs), the development of advanced computational algorithms, and access to vast amounts of data from various data sources in relevant domains ^3,4^. This has facilitated standard, systematic benchmarking of AI models across domains, which has revolutionized machine learning and deep learning (ML/DL), including computer vision and natural language processing, with successful applications in areas as diverse as self-driving cars and face and speech recognition ^5–9^. Similarly, the widespread adoption of electronic health records (EHRs) in healthcare has substantially increased the amount of digital clinical data, available for data-driven analysis. Nevertheless, the application of ML in healthcare research encounters various hindrances, including ethical, security, and privacy concerns regarding patient data, which restrict researchers’ access to pertinent datasets ^10–12^. Consequently, many studies have utilized unpublished data without external accessibility, rendering them unsuitable for benchmarking ML models. Despite regularly reporting state-of-the-art performance, confirming claims of model superiority remains a challenge ^13^. Approaches like distributed or federated learning can mitigate privacy risks by keeping data localized and not openly shared^14,15^. However, they still limit data to algorithms and restrict their usefulness in certain scenarios, such as independent verification by researchers or training custom models for specific applications. These issues highlight the crucial need to openly publish medical datasets to a broader audience while preserving patient data privacy. Such measures are imperative for more accurate and standard model benchmarking, ensuring transparency and reproducibility in healthcare research.

The intensive care unit (ICU) has been at the forefront of ML modeling for assisting in clinical decision support, largely because of the wealth of data available from EHRs ^16,17^. Numerous studies have utilized ICU data to develop ML/DL models for critical prediction tasks in healthcare, such as early detection of medical complications like heart failure, acute kidney injury, or sepsis, as well as prediction of care outcomes including length of stay (LOS) in the ICU or readmission to the ICU (hospital) ^17–19^. The latter is important since outcomes such as prolonged ICU (hospital) stays or high readmission rates can place a significant financial burden on healthcare institutions. The COVID-19 pandemic has underscored the necessity of dependable ML-based clinical decision support to aid in clinical diagnosis, suggest treatment options, and improve hospital resource allocation and management ^20–22^.

Publicly available datasets are being widely used in critical care research, specifically for training and validating ML models. These datasets have become essential components for advancing healthcare research and services, ultimately improving patient outcomes ^23^. The Medical Information Mart for Intensive Care (MIMIC) released the MIMIC-II database in 2011, serving as the first publicly available single-center ICU database ^24^. The MIMIC database has undergone several updates and has been the subject of extensive studies. However, since the original release of the MIMIC database, few other adult ICU databases have been made accessible for research purposes ^23^. These include the U.S. multicenter ICU database, namely the eICU Collaborative Research Database (eICU-CRD), and two European single-center ICU databases; the Amsterdam University Medical Center database (AUMCdb), and the High Time Resolution ICU Dataset (HiRID) ^25–27^. A new ICU database, the Salzburg Intensive Care database (SICdb), was released by the University Hospital Salzburg in Austria in early 2023 ^28–30^. The present work aims to analyze the data provided in SICdb (version 1.0.6) and compare its characteristics to those of the well-established MIMIC dataset. The MIMIC provides a rich repository of clinical data across diverse patient populations and medical conditions in the ICU and emergency department. It has been extensively used in ICU analytics and data-driven healthcare research ^31–34^, and also has been the subject of previous studies for similar comparative analysis, demonstrating its utility and reliability in clinical research ^23^. This study utilized MIMIC-IV version 2.1, which was published in November 2022 and included patients admitted to the BETH Israel Deaconess Medical Center between 2008 and 2019. The next section presents a brief description of the SICdb and its structure, followed by a discussion of the temporal behavior of the data and the distribution of diagnoses. The paper concludes by briefly examining the impact of COVID-19 on certain care outcomes.

## SICdb

**Figure 1:**
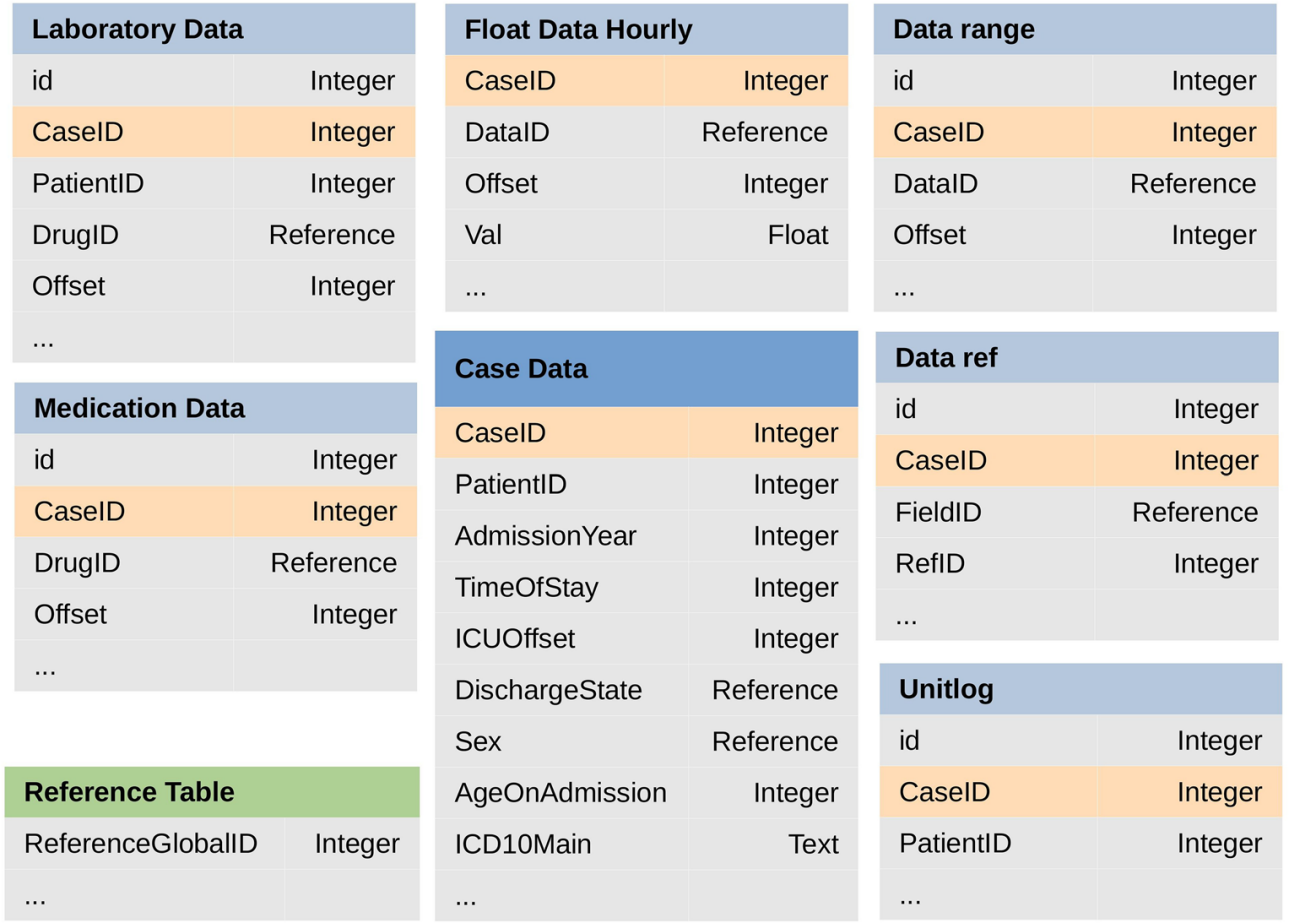
The data in SICdb are organized into several data tables, shown in blue. These tables are linked to the main table (‘Case Data') through a key identifier (*CaseID*). The data type of each feature is indicated as Integer, Float, or Reference. The ‘Reference Table’, shown in green, provides information about encoded data fields in the SICdb.

The SICdb is a data repository comprising data from four ICUs at the University Hospital Salzburg, collected from 2013 to 2021 by the Salzburger Landesklinik (SALK) - Department of Anesthesiology and Intensive Care Medicine, and the Paracelsus Medical University of Salzburg (PMU). The data in the SICdb have been anonymized in accordance with article 4(5) of the European General Data Protection Regulation (GDPR) ^28^. The database includes data exclusively from ICU patients and consists of seven data tables with different characteristics. The data tables are illustrated in blue in Figure 1, along with some example features and key identifiers in each. The primary table, referred to as ‘Case Data’ (cases), supplies the baseline characteristics of patients upon admission to the ICU. Patient demographics and admission data, such as age, sex, year of admission, length of stay, diagnosis, and numerous other variables, were collected in this table. Additional data tables provide various detailed information for different purposes. The ‘Laboratory Data’ (laboratory) table includes measurements, values, units, and the recording time of laboratory tests performed during the patient’s stay in the ICU. The ‘Medication Data’ (medication) table contains medication information, including drug codes, dosing times, and dosages administered to individual patients. The largest data table is the ‘Float Data Hourly’ (data_float_h), which offers extensive details about the time and values of various vital signs. This table presents minutely collected signal data encoded in binary format, along with an hourly aggregated values calculated using the arithmetic mean. The ‘Data range’ (data_range) table provides ranged data on treatment or invasive procedures, such as central lines or drainages, while the ‘Data ref’ (data_ref) contains encoded (nominal) data unique to each admission. Finally, the ‘Unitlog’ (unitlog) provides details on patient transportation and stay. Each of these tables can be linked to the ‘Case Data’ through a unique identifier (CaseID). The ‘Reference Table’ (d_reference) in green contains information about encoded data fields in the SICdb, facilitating the translation of codes into the actual names of all vital signs, laboratory data, medications, etc. Additional information is available in the source documentation (https://www.sicdb.com/Documentation/Main_Page, accessed on 24 January 2024).

### Data description

**Figure 2:**
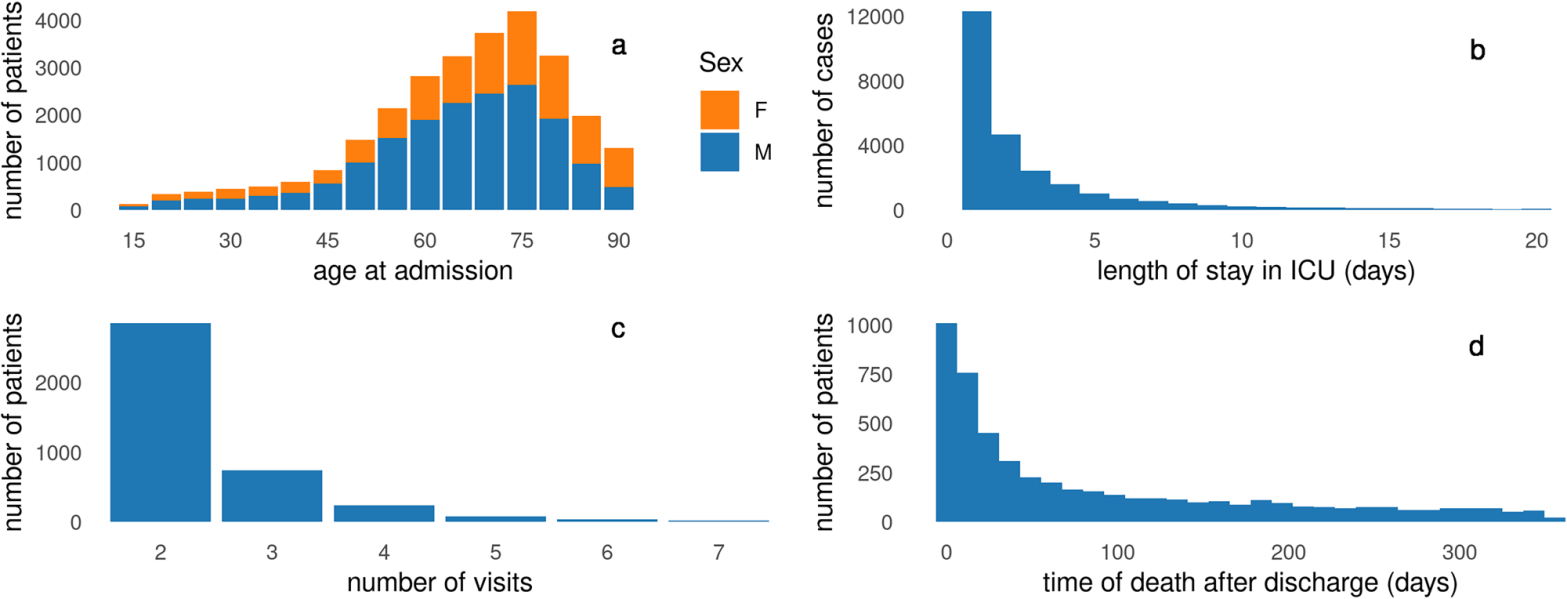
The SICdb data characteristics: (**a**) the age distribution for both genders (F: female, M: male), (**b**) distribution of LOS in the ICU for less than three weeks (for visualization), (**c**) distribution of patients with multiple ICU visits up to seven times (for visualization), and (**d**) distribution of time of death up to one year after discharge, when it is known.

The SICdb contains a total of 27,386 admissions for 21,583 patients, consisting of 13,371 (62.0%) males and 8,212 females. To protect privacy in the SICdb, patient age was grouped into 5-year intervals. The mean age was 65.67 years with standard deviation (SD) of 16.16 years. Figure 2a displays the age distribution for both genders. The LOS in the ICU (the period between admission to the ICU and discharge to the ward) was measured in seconds. On average, patients stayed in the ICU for 3.5 days with a SD of 6.5 days and a maximum stay of approximately 207 days. As is typical, the LOS distribution is left-skewed with a long tail on the right side, as shown in Figure 2b. Additionally, there is another time period called *ICUOffset* which refers to the time a patient spent in the operation unit while already registered in the ICU. In most cases, this occurs before admission to the ICU, as noted in the original paper ^28^. Although subtracting the *ICUOffset* from the LOS does not significantly alter the mean LOS, it still has some impact. Among the ICU patients, 17,597 (81.5%) were admitted once, while 3986 were admitted multiple times, with up to 12 visits. For illustrative purposes, Figure 2c excludes patients with a single admission and more than seven visits. The ICU experiences approximately 3043 admissions per year, peaking in 2017 with 3586 admissions. If the time of death was known, the data were tracked up to one year after ICU discharge (Figure 2d). The SICdb records a total of 5093 deaths, with 1139 (21.85%) of which occurring during the patient’s stay. The overall mortality rate for all patients was approximately 5.2%, and the mortality rate after 28 days was 9.9%.

### Comparison with the MIMIC-IV database

To gain a closer understanding of the SICdb, we contrast it with the MIMIC-IV database, which has been widely used in medical data science for various purposes ^31–34^. To this end, we will use MIMIC-IV version 2.1, which is accessible on PhysioNet.org under specific conditions. The key distinction is that the MIMIC-IV not only focuses on ICU data, but also encompasses other available hospital data, while the SICdb exclusively concentrates on ICU data. Both databases are single-center and cover a period of 9 years in SICdb (2013-2021) and 12 years in MIMIC-IV, with 7 years of overlap. ICU data are influenced by historical global events such as the COVID-19 pandemic or local events that may pose challenges for healthcare systems, hospital staff, and patients. The SICdb has a lower number of ICU patients (21,583) than the MIMIC-IV (50,934), yet the average LOS is comparable in both datasets at 3.50 and 3.46 days, respectively. The average age of patients in the SICdb was greater than that in the MIMIC-IV (66 versus 65 years), with 6% more male patients in the SICdb. Table 1 presents a summary of the statistical comparisons between the two databases.

**Table 1:** Comparison of ICU patient characteristics and outcomes in two single-center databases, SICdb and MIMIC-IV.

Characteristics	SICdb	MIMIC-IV
Center location	Salzburg, Austria	Boston, United States
Time period	2013–2021	2008–2019
ICU unique patient counts	21,583	50,934
ICU patient age: median (IQR)	66 (55–75)	65 (53–77)
Gender: male	62.0%	56.0%
Mortality: ICU	5.2%	7.7%
Mortality: 28 d	9.9%	15.2%
LOS in ICU: median (IQR)	1.5 (1.0–3.3)	1.9 (1.1–3.7)
Patients with $>=1$ comorbidity	47.6%^†^	83.8%^‡^
Severity of illness scores	SAPS III	APACHE III, SOFA, OASIS
Availability of radiology images	Not reported	Yes
Availability of clinical notes	Not reported	No

^†^ Requires detailed information with specific data.

${\ddag }$ Considering ICD codes of version 10.

It is noted that the comorbidity was calculated differently in the SICdb due to limited data availability, with only primary diagnoses being reported. It should also be noted that the SICdb reports the SAPS III (Simplified Acute Physiology Score) for severity of illness, while MIMIC-IV provides APACHE (Acute Physiology and Chronic Health Evaluation), SOFA (Sequential Organ Failure Assessment), as well as OASIS (Oxford Acute Severity of Illness Score). Additionally, the current version of SICdb does not provide radiology images or clinical notes. The interquartile range (IQR) represents the second and third quartiles, indicating the variability of data.

**Figure 3:**
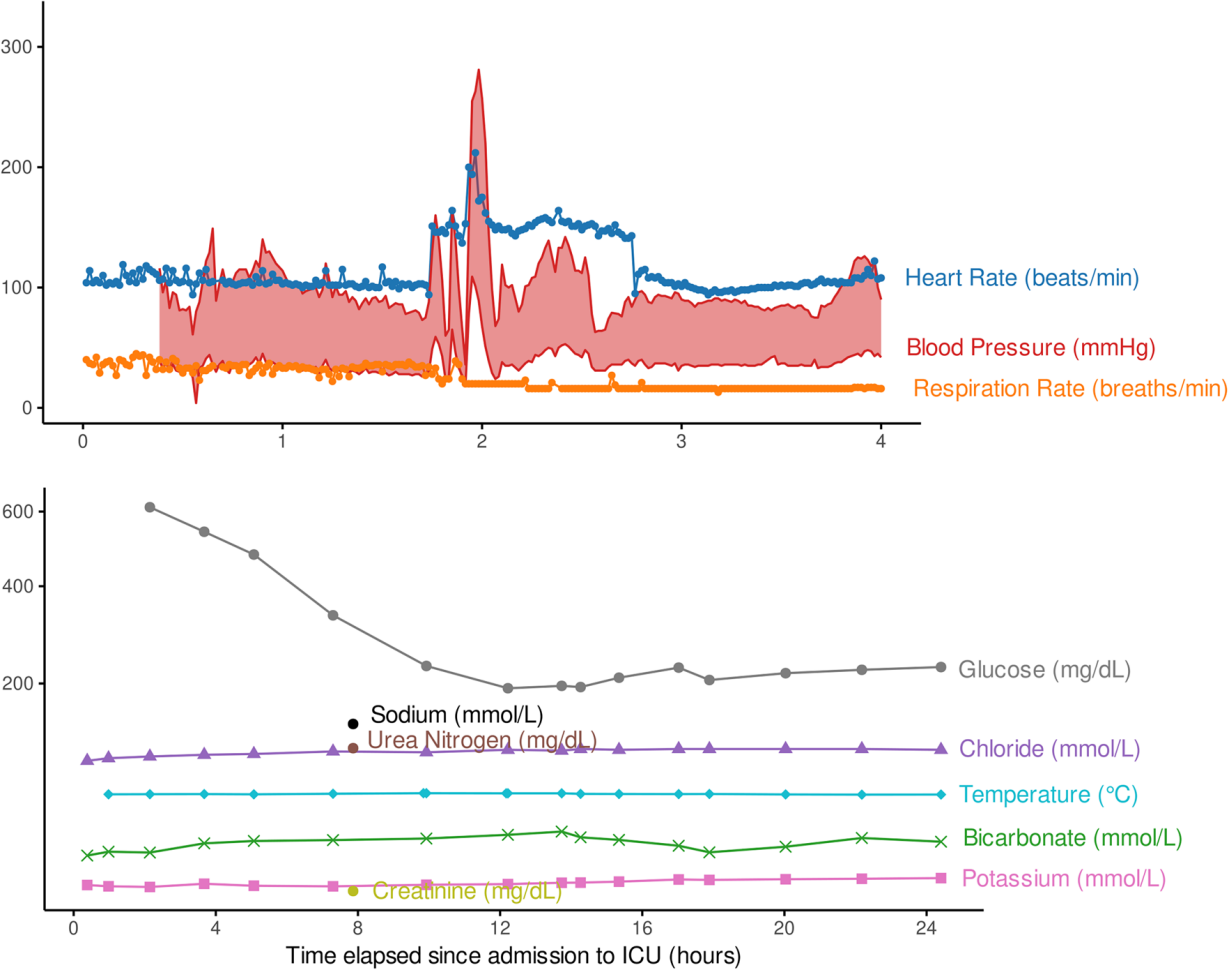
Time series ICU data from a patient with *patientID* 406214 and a LOS of 18.71 days in the SICdb. The upper panel displays some vital signs only for the first four hours after admission because of their high temporal resolution. The lower panel represents laboratory measurements for the full initial 24 hours of the patient’s stay in the ICU.

### Time series health data-vital signs and laboratory measurements

During hospitalization, health data such as vital signs and laboratory measurements are often recorded multiple times. Time series health data are valuable for understanding the longitudinal trajectory of patients’ health and ailments, as they contain important information concealed in their corresponding features ^35,36^. The effective use of time series data can greatly enhance the progress of data-driven ML in healthcare research. However, this aspect has not received sufficient attention to date, mostly due to the limited availability of time series data. The SICdb delivers high-resolution time series data recorded with one-second granularity. Figure 3 shows the time series data for several vital signs and laboratory measurements in the SICdb during the first day after ICU admission. Only the first four hours of vital signs are presented due to their high temporal resolution. To facilitate a comprehensive understanding of the temporal characteristics of the SICdb, its time series data are compared with those of the MIMIC-IV. It is important, however, to distinguish between machine-generated data, which often include automatically collected vital signs such as heart rate, blood pressure, or oxygen saturation, and non-machine-generated data, which must be requested by the physician and depend on the patient’s condition or specific events.

The SICdb contains approximately 1.5 billion entries for vital signs, compared to only 314 million entries in the MIMIC-IV dataset. This suggests that patient data were more frequently recorded throughout hospitalization in the SICdb than in the MIMIC-IV. It is also worth noting that the SICdb covers 60 different items for vital signs, while the MIMIC-IV includes 50 items. With 124,867,711 entries across 21,583 admissions, the SICdb provides the highest number of records for *heart rate ECG*, while the MIMIC-IV dataset contains 6,463,819 heart rate records from 66,187 admissions and has the largest count for the *safety measures*, with a total of 8,783,217 entries from 67,840 admissions. To ensure a fair comparison between the two datasets, we evaluated the availability and frequency of vital signs and laboratory data during the initial 24 hours after admission in both datasets. Heart rate, respiratory rate, and oxygen saturation (SpO2) are the most commonly recorded vital signs in the ICU for both datasets, and have the highest coverage among all ICU patients. In the SICdb, invasive blood pressure is the next most prevalent variable, while in the MIMIC-IV, non-invasive blood pressure (systolic, diastolic, and mean) is slightly more prevalent than invasive blood pressure.

**Figure 4:**
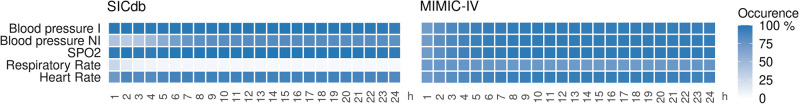
Availability of most common vital signs for patients with LOS longer than one day, during the initial 24 hours of their ICU stay in both the SICdb and MIMIC-IV. The displayed squares represent the percentage of the available data for each stay, partitioned over the 24 hours of the first day in the ICU.

The availability of the most common vital signs was analyzed in both datasets during the first day of stay in the ICU. Figure 4 displays the number of unique stays for vital signs binned in one-hour intervals over the initial 24 hours following admission. Blood pressure, invasive heart rate, and SpO2 in the SICdb exhibit similar trends over the first 24 hours, while non-invasive blood pressure occurs less than 30% and rapidly diminishes. The respiratory rate is less frequent during the first four hours, but gradually increases thereafter. These parameters are available for more than two-thirds of all ICU patients in the MIMIC-IV dataset, and they exhibit a consistent or increasing trend. Within the first 24 hours, the SICdb provides data on heart rate, SpO2, and invasive blood pressure for more patients than does the MIMIC-IV database. However, respiratory rate and non-invasive blood pressure data are less available in the SICdb during the first day of stay.

The frequency of measuring and recording medical variables over time is another noteworthy aspect of time series ICU data. We calculated the average vital sign measurements per hour to determine the frequency of occurrence. To calculate this, simply divide the number of entries for a specific parameter by the LOS (measured in hours) in the ICU. Table 2 displays the results for several vital signs in both datasets. We used the same parameters as those selected for data availability to ensure comparability. The values indicate that the SICdb database has a significantly greater occurrence frequency compared to the MIMIC-IV, except for non-invasive systolic blood pressure. For instance, the average number of heart rate measurements per hour is 56.8 in the SICdb, while the MIMIC-IV records only 1.09 measurements per hour. The high frequency of the SICdb measurements is evident for some vital signs, with most measurements taken almost once per minute, as they are largely generated by machines. This frequency surpasses even that of the HiRID database, as reported by *Sauer et al*^23^.

**Table 2:** Comparison of the mean frequency of vital signs in the SICdb and MIMIC-IV.

	SICdb	MIMIC-IV
Heart rate	56.8	1.09
Respiratory rate	48.3	1.08
Oxygen saturation (SpO2)	55.2	1.06
Blood pressure systolic invasive	51.4	0.75
Blood pressure systolic non-invasive	1.14	0.78

The values represent the average number of measurements per hour for each vital sign during the entire ICU stay.

Laboratory measurements are also recorded several times throughout the ICU stay, but at a lower frequency than vital signs. Analyzing the temporal behavior of laboratory parameters is more challenging due to inconsistencies between the two datasets, SICdb and MIMIC-IV, when mapping different items. For instance, the Logical Observation Identifiers Names and Codes (LOINC), a universal standard for identifying medical laboratory parameters, is not reported in the SICdb. The MIMIC-IV also has a significantly lower coverage of LOINC, at only 2.1%, compared to MIMIC-III, which covers 77.7% of the laboratory parameters. Additionally, potential variations in measurement methods during the observed period, which may not be available to the data users, could lead to difficulties.

**Figure 5:**
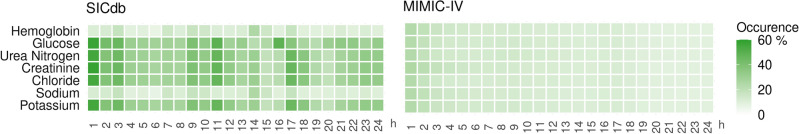
Availability of most frequent laboratory measurements for patients with LOS longer than one day, during the initial 24 hours of their ICU stay in both the SICdb and MIMIC-IV. The displayed squares represent the percentage of the available data for each stay, partitioned over the 24 hours of the first day in the ICU.

For data representation, we selected the top ten laboratory parameters with the highest occurrence rate across all patients in each dataset. We then attempted to match the item labels based on the available patient data, measurement unit, and type of extracted tissue for medical tests. The SICdb incorporates a greater amount of time series data that does the MIMIC-IV, as evidenced by the larger number of laboratory measurements. Figure 5 provides evidence of a higher incidence of all laboratory parameters during the first 24 hours after ICU admission in the SICdb than in the MIMIC-IV database. However, the analysis of the SICdb and MIMIC-IV revealed only marginal differences in the frequencies of some laboratory parameters, such as *urea nitrogen* (12.7% in SICdb compared to 12.6% in MIMIC-IV) and *creatinine* (13.1% in SICdb compared to 12.6% in MIMIC-IV), with a slightly higher incidence rate in the SICdb.

Differences in occurrence patterns between the two datasets are observed. The SICdb includes laboratory measurements taken at specific hours after admission, such as 0-1, 10-11, and 16-17 hours, suggesting a probable routine monitoring of laboratory values by clinical personnel. In contrast, the MIMIC-IV dataset contains consistently low numbers of available data points for all ICU patients. However, this analysis excludes certain challenges inherent in time series data in both datasets. For instance, we analyzed the duration of time after admission, considering the 24-hour day-night cycle, which cannot be measured in the SICdb.

### Diagnoses

**Figure 6:**
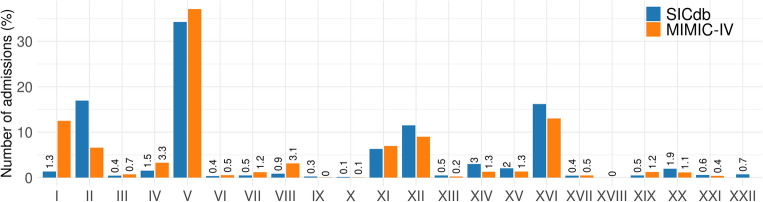
The distribution of primary diagnoses in the SICdb compared to that found in the MIMIC-IV. The list of 22 disease chapters (I ... XXII) can be found in the Supplementary Material for reference.

The SICdb represents diagnoses using ICD-10 codes, with only the primary diagnosis being accessible. Secondary diagnoses can be obtained upon request to SICdb providers^28^. Currently, the SICdb has 27,295 available diagnoses with 2169 unique ICD-10 codes. The most frequently occurring diagnosis, with 1883 instances, is *I25.1*, which corresponds to atherosclerotic heart disease. For analysis purposes, we have grouped the diagnoses into 22 chapters of the ICD-10 as defined by the World Health Organization (WHO) (https://icd.who.int/browse10/2019/en). The list of chapters can be found in the Supplementary Material for reference. A diagnosis code that cannot be identified was categorized as NA. The distribution of primary diagnoses in the SICdb appears comparable to that found in the MIMIC-IV, as shown in Figure 6. The most common diagnoses are related to diseases of the cardiovascular system (chapter IX), injuries and poisonings (chapter XIX), diseases of the digestive system (chapter XI), and diseases of the respiratory system (chapter X). The remaining diagnosis groups have a smaller number of cases and represent minorities. Compared to MIMIC-IV, *neoplasms* are more highly represented in chapter II of the SICdb. However, it is important to note that the incidence of infections and parasitic diseases is higher in MIMIC-IV than in the SICdb. Furthermore, the MIMIC-IV does not include any primary diagnoses from chapter XXII (codes for special purposes). To analyze MIMIC-IV diagnoses, we selected only those admissions that visited the ICU and considered the first diagnosis listed in the ICD-10 codes as the primary diagnosis. This approach reduced the number of ICU admissions to 29,656 cases. We note that in the MIMIC-IV, each stay may have multiple diagnoses given by ICD codes, and the first one listed may not necessarily be the primary diagnosis(https://mimic.mit.edu/docs/iv/modules/hosp/diagnoses_icd/.).

### Impact of COVID-19 on care outcomes

The SICdb covers data from the onset of the COVID-19 pandemic in late 2019, while the MIMIC-IV incorporates data only up to 2019, largely unaffected by the pandemic’s emergence. To identify patients with COVID-19, we relied on the ICD-10 coding system documented for diagnoses in the SICdb, despite its inherent limitations. The official ICD-10 classification for COVID-19 was introduced in February 2020(https://www.who.int/standards/classifications/classification-of-diseases/emergency-use-icd-codes-for-covid-19-disease-outbreak.).

This temporal gap between the initial outbreak of COVID-19 in late 2019 and its official classification is noteworthy. Moreover, only patients diagnosed with COVID-19 as their primary condition were included, given that the SICdb exclusively provides primary diagnoses. The SICdb recorded 186 instances of COVID-19 for 180 patients with an ICD-10 code of *U07.1* *‘virus identified’*, comprising 83 admissions in 2020 and 103 in 2021. It is worth noting that the ICD-10 code for COVID-19 with *‘not identified virus’* (*U07.2*) was not considered, as it is not listed as a primary diagnosis in the SICdb. The median LOS for these COVID-19 patients is 6.92 days (with a mean of 12.72 days), almost five times higher than the average LOS for the entire dataset. Patients primarily diagnosed with COVID-19 accounted for less than 1% of the overall patient population, representing 3.19% in 2020 and 3.75% in 2021, thereby exerting minimal influence on the total LOS. It should also be noted that identifying patients with secondary COVID-19 diagnosis is not a straightforward task. For instance, considering patients with both Pneumonia and SarsCoV2 could add 47 more patients to the analysis, yet these patients were excluded from the consideration.

When comparing the care outcomes of the period of 2020-2021, during which COVID-19 was present, to the years 2013-2019 without COVID-19, there appears to be a small differences. For instance, the LOS is slightly longer during the COVID-19 period (3.61 days) than during the non-COVID-19 period (3.47 days). Additionally, during the COVID-19 period, the number of readmitted patients was lower (702 patients) compared to the non-COVID-19 period (3411 patients), with some patients overlapping in both periods. However, there were some differences in the distribution of diagnoses between the COVID-19 period (2020 and 2021) and the non-COVID-19 period. In particular, approximately 200 fewer cases of diseases of the cardiovascular system (XI) were diagnosed per year during the COVID-19 period. This could be attributed to the greater occupancy of the ICU by COVID-19 patients, yet further investigation is needed to confirm this correlation.

## Conclusions

As the application of AI in medicine advances, there is a growing need for publicly available data to benchmark ML/DL models. The recent release of the SICdb, a new dataset from Austria, offers researchers around the world more diverse and extensive insight into available medical data. This study presents a concise overview of the dataset’s characteristics and compares it to the well-established MIMIC-IV dataset. The structure of the ICU section in the MIMIC-IV database is comparable to SICdb. However, MIMIC-IV separates patient data from the cases (stays) that are more relevant to patient care ^37^. To ensure the reliability of our comparative analysis, we limited the comparison to the consistent parts of the two databases. To this end, we conducted a thorough exploration and examination of the data from both databases to identify any potential contradictions or gaps that could impact the analysis. We note that any variations in data collection methodologies can greatly impact the comparability of datasets. When comparing measurements, it is crucial to consider the names, units, data ranges, and appearances in the dataset to ensure consistent parameters. For instance, in SICdb, heart rate is measured using ECG (originally referred to as ‘heartRateEKG’), while in MIMIC-IV, heart rate is measured as part of routine vital signs using the same method.

The SICdb provides high-resolution temporal data that are more suitable for longitudinal studies of patients’ health conditions, in comparison to the MIMIC-IV. This feature can, for example, facilitate more accurate yet complex modeling of care outcome predictions, such as patient readmission to the ICU or care acuity, using long short-term memory (LSTM)^38,39^. High resolution time series data can also provide a better understanding of the patient’s pathway in the ICU and assist in predicting adverse events. We believe that that both data scientists and the intensive care community will benefit from high-resolution ICU datasets that include pre- and post-discharge data, quality of life measures, and pre- and post-ICU hospital data. It should be however noted that the suitability of a database may vary depending on the specific domain of application and the research questions being asked.

Furthermore, the SICdb contains data on ICU patients during the COVID-19 pandemic, a worldwide adverse that may affect patient care data during this time. A preliminary comparison to the MIMIC-IV revealed that patients diagnosed with COVID-19 has a significantly longer LOS. However, unlike the MIMIC-IV, the SICdb only includes primary ICD codes, making it difficult to identify patients diagnosed with COVID-19 as a secondary disease. This fact also limits the ability to calculate certain scores, such as comorbidity scores. Additionally, the current version of the SICdb does not provide image data and clinical notes. Despite these limitations, the publication of the SICdb represents a significant advancement in the provision of diverse public medical data, particularly for the ICU. This will undoubtedly facilitate the development of AI applications in healthcare.

### Supplementary Information

Supplementary Information.

## Data Availability

The data that support the findings of this study are available from PhysioNet, but restrictions apply to the availability of these data, which were used under license for the current study, and so are not publicly available. Data are however available from the authors upon reasonable request and with permission of PhysioNet.

## Code availability

All source codes used for data access and analysis are publicly available at https://gitlab.com/ul-mds/data-science/overview_sicdb. For this study, we utilized SICdb version 1.0.6 and MIMIC-IV version 2.1.
