# Supplementary material for: Salzburg Intensive Care database (SICdb): a detailed exploration and comparative analysis with MIMIC-IV
Source: Sci Rep. 2024 May 20;14:11438. doi: 10.1038/s41598-024-61380-0 (PMC11102905; doi:10.1038/s41598-024-61380-0)
Supplement: Supplementary file 1 — Supplementary Information. [file 41598_2024_61380_MOESM1_ESM.pdf]

Supplementary Material

This section summarizes the list of the 22 chapters of ICD-10 codes version 2019 that indicate groups of specific diseases, morbid conditions, symptoms, signs, and abnormal clinical and laboratory findings, including those not classified elsewhere, as well as injuries, poisonings, and other external causes of morbidity and mortality. The final two chapters address the factors that affect health status and contact with health services, as well as the provisional classification of new diseases with unknown causes.

I	Certain infectious and parasitic diseases
II	Neoplasms
III	Diseases of the blood and blood-forming organs and certain disorders involving the immune mechanism
IV	Endocrine, nutritional and metabolic diseases
V	Mental and behavioural disorders
VI	Diseases of the nervous system
VII	Diseases of the eye and adnexa
VIII	Diseases of the ear and mastoid process
IX	Diseases of the circulatory system
X	Diseases of the respiratory system
XI	Diseases of the digestive system
XII	Diseases of the skin and subcutaneous tissue
XIII	Diseases of the musculoskeletal system and connective tissue
XIV	Diseases of the genitourinary system
XV	Pregnancy, childbirth and the puerperium
XVI	Certain conditions originating in the perinatal period
XVII	Congenital malformations, deformations and chromosomal abnormalities
XVIII	Symptoms, signs and abnormal clinical and laboratory findings, not elsewhere classified
XIX	Injury, poisoning and certain other consequences of external causes
XX	External causes of morbidity and mortality
XXI	Factors influencing health status and contact with health services
XXII	Codes for special purposes
